# Genetic Variability and Structuring of Arctic Charr (*Salvelinus alpinus*) Populations in Northern Fennoscandia

**DOI:** 10.1371/journal.pone.0140344

**Published:** 2015-10-15

**Authors:** Takahito Shikano, Antero Järvinen, Paula Marjamäki, Kimmo K. Kahilainen, Juha Merilä

**Affiliations:** 1 Ecological Genetics Research Unit, Department of Biosciences, University of Helsinki, Helsinki, Finland; 2 Kilpisjärvi Biological Station, University of Helsinki, Kilpisjärvi, Finland; 3 Department of Environmental Sciences, University of Helsinki, Helsinki, Finland; University of Iceland, ICELAND

## Abstract

Variation in presumably neutral genetic markers can inform us about evolvability, historical effective population sizes and phylogeographic history of contemporary populations. We studied genetic variability in 15 microsatellite loci in six native landlocked Arctic charr (*Salvelinus alpinus*) populations in northern Fennoscandia, where this species is considered near threatened. We discovered that all populations were genetically highly (mean *F*_ST_ ≈ 0.26) differentiated and isolated from each other. Evidence was found for historical, but not for recent population size bottlenecks. Estimates of contemporary effective population size (*N*_e_) ranged from seven to 228 and were significantly correlated with those of historical *N*_e_ but not with lake size. A census size (*N*_C_) was estimated to be approximately 300 individuals in a pond (0.14 ha), which exhibited the smallest *N*_e_ (i.e. *N*_e_/*N*_C_ = 0.02). Genetic variability in this pond and a connected lake is severely reduced, and both genetic and empirical estimates of migration rates indicate a lack of gene flow between them. Hence, albeit currently thriving, some northern Fennoscandian populations appear to be vulnerable to further loss of genetic variability and are likely to have limited capacity to adapt if selection pressures change.

## Introduction

Evolutionary adaptation to abiotic and biotic selection pressures is fuelled by genetic variability. Low genetic variability, or lack thereof, can reduce the rate or even prevent adaptation. Reduced genetic variability is common in small and isolated populations, such as many freshwater fishes landlocked in discrete lakes and ponds (e.g. [1,2]). The reduced genetic diversity in small populations is chiefly attributable to their small effective population size promoting erosion of genetic variability due to genetic drift, inbreeding and lack of gene flow [3]. However, due to historical population size bottlenecks, contemporarily large populations may also have low genetic variability, as is the case in many post-glacially established populations in northern Europe [4,5].

Although there has been considerable debate around the informativeness of (presumably) neutral marker genes–such as microsatellites–as indicators of populations’ adaptive potential (e.g. [6] and references therein), recent theoretical treatments suggest that marker variability is indeed informative about adaptability [7–9]. Hence, closed populations with low genetic variability are expected to be vulnerable to maladaptation in the face of changing environmental conditions, such as those brought on by climate change, habitat transition and invasive species [10].

The Arctic charr (*Salvelinus alpinus*) is the northernmost freshwater fish in the world, with a circumpolar distribution [11,12]. It is a phenotypically and ecologically diverse cold-water fish occurring in lakes, ponds, rivers and coastal areas of low salinity [12,13]. A large-scale phylogeographic study has uncovered five ancestral lineages in the Holarctic region derived from different glacial refugia [14]. The species has been subject to numerous population genetic studies in different parts of its distribution range (e.g. [15–24]). These studies have typically revealed a relatively high degree of population structuring, and often also reduced levels of genetic variability in landlocked populations as compared to anadromous populations (e.g. [17,19,22–24]). Despite the abundance of this species in Scandinavia [25], two southern Finnish fringe populations are currently classified as critically endangered (CR), and the northern Finnish populations are considered near threatened (NT) [26]. Hence, assessments of genetic diversity, degree of isolation and effective population sizes are required for the conservation of this species. In addition, for a better understanding of the underlying causes of the low genetic variability and high population subdivision which are often found in this species, it is important to assess the relative impact of historical and contemporary factors on the current genetic diversity.

**Fig 1:**
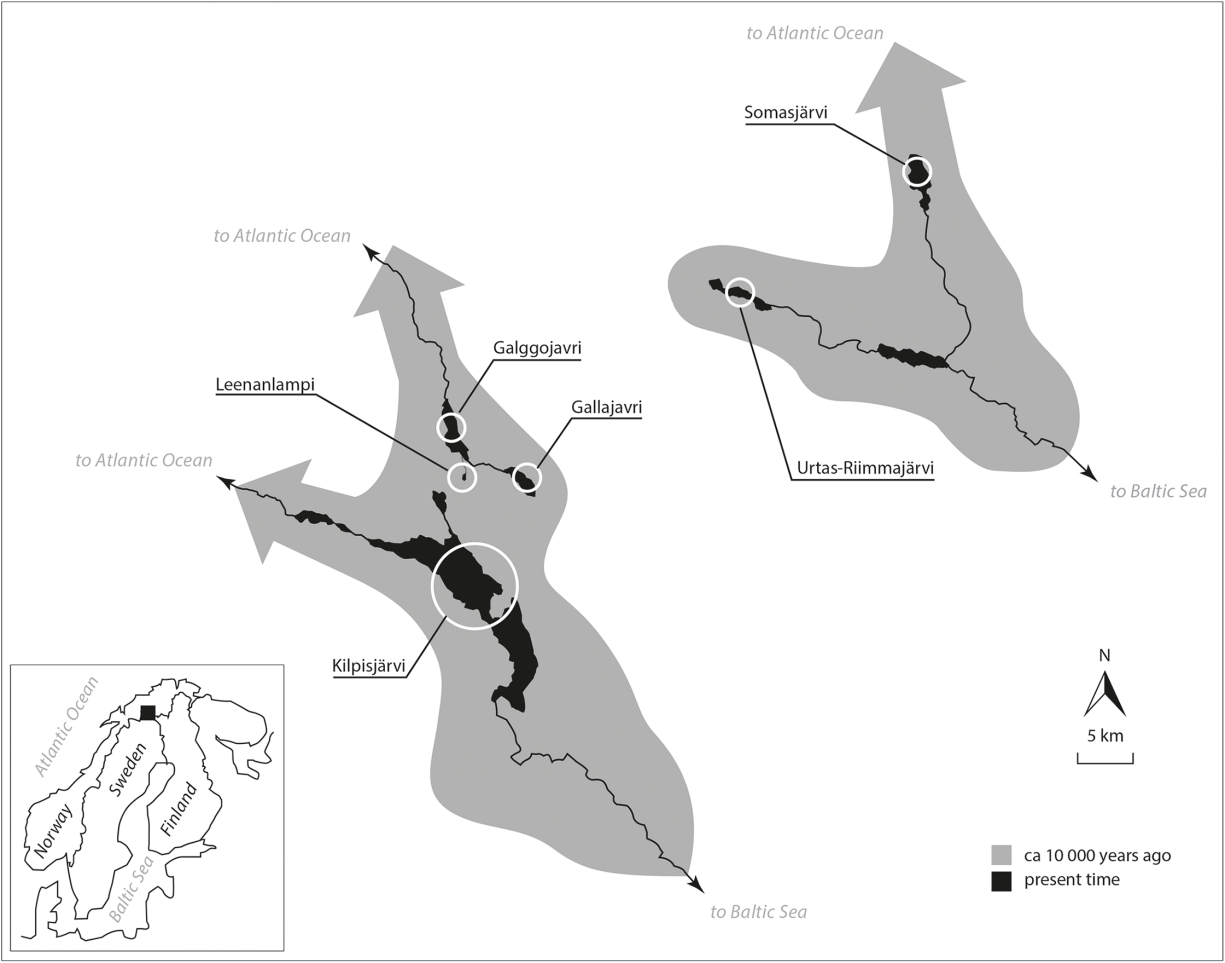
Sampling locations of the six Arctic charr populations. The current situation is presented in black, in which Lakes Kilpisjärvi, Urtas-Riimmajärvi and Somasjärvi belong to the watercourse draining into the Baltic Sea and the other lakes belong to the watercourse draining into the Atlantic Ocean. Gray shading indicates the historical (ca. 10 000 years ago) situation, when all lakes were involved in two separate watercourses draining into the Atlantic Ocean. All studied populations are landlocked at the present day.

The aim of this study was to investigate genetic variability and population structuring of Arctic charr in northern Fennoscandia where two watersheds meet: three of the study populations reside in lakes draining into the Baltic Sea, while three occupy two lakes and a pond draining into the North Atlantic Ocean (Fig 1). Some of the lakes and pond are connected by pristine rivers and brooks, but given the complex post-glacial history of melting ice sheets and large ice-dammed lakes in this area [27–30], it is not clear how genetic diversity is distributed and maintained within and among the populations. We assessed the degree of genetic independence among the different populations, as well as looked for evidence of past and recent population size bottlenecks. In addition, historical and contemporary effective population sizes were estimated in order to assess the vulnerability of the populations to further loss of genetic diversity. We were particularly interested in effective population size and genetic variability in a very small (0.14 ha) pond population for which census population size estimates, as well as migration rate estimates, were assessed from field data.

## Materials and Methods

### Ethics statement

This study was performed in strict accordance with the Finnish and Norwegian legislation. Fishing rights in Finland belong to the land owner according to the Finnish Fishing Law (5§ 27.5.2011/600). Accordingly, the fishing permits were obtained from the land owner, Finnish Forest and Park Service (permit numbers 3221–3240, 14.6.2010, 14.2.2011, 31.1.2012). Fish were euthanized by cerebral concussion for tissue collection immediately after their capture in accordance with the Finnish Animal Conservation Law (32§9.8.2013/584). No ethical permission is required for described scientific sampling with gill nets according to the Finnish Animal Conservation Law (7§ 28.6.2013/498). As to the sampling of fish from the Norwegian localities, a fishing permission is required from the fishing right owner. Accordingly, we obtained permissions for the gill net fishing in Galggojavri and Leenanlampi from the County Governor of Troms (permission numbers: 10/659-6, 28.4.2010, 10/659-23, 26.4.2011 and 10/659-52, 30.4.2012) with legal authority through LOV 1992-05-15 nr 47, §13. No ethical permission is required from the Norwegian Animal Research Authority for sampling and described activities (FOR 1996-01-15 nr 23, the Norwegian Ministry of Agriculture and Food).

### Study populations and sampling

The six Arctic charr populations included in this study were collected at a watercourse divide: two lakes (Galggojavri and Gallajavri) and one pond (Leenanlampi) in the Skibotn watercourse drain into the Atlantic Ocean and three lakes (Somasjärvi, Urtas-Riimmajärvi and Kilpisjärvi) in the Tornio-Muoniojoki watercourse drain into the Baltic Sea (Fig 1). The lakes and pond within each of the two watercourses are connected by rivers, but steep rapids and long distances separate them (Fig 1). Spatial location, lake morphometry and sample size (average ≈ 55 individuals) for each locality are given in Table 1. Most of the samples were collected using monofilament gillnets (mesh sizes, 12–60 mm); in two cases (Leenanlampi and Urtas-Riimmajärvi) rod fishing and wire traps were also used. Sampling was done in June–September 2010–2012, except that Urtas-Riimmajärvi was sampled in April–May of 2012. A piece of dorsal muscle or adipose fin tissue was collected from each fish and preserved in ethanol for later DNA extraction. All captured fish from Leenanlampi were returned alive to the pond soon after small adipose fin clips were taken. An earlier study of Arctic charr genetics in Finland [18] has sampled three lakes in this region including Lake Somasjärvi, but the focus was on hatchery versus native Arctic charr comparisons. The localities included in the present study host only native fish: none of the sampled locations are known to be subject to stocking or farming activities.

**Table 1:** Study sites and genetic variation at 13 microsatellite loci in the six Arctic charr populations.

Habitat type		Site			Genetic variation					
	Population	Coordinates	Altitude (m)	Lake size (ha)	*N*	*Ar*	Private *Ar*	*H*_E_	*H*_O_	*F*_IS_
Lake										
	Somasjärvi	69° 17' N, 21° 32' E	732	181	50	6.7	0.4	0.640	0.592	0.063
	Urtas-Riimmajärvi	69° 12' N, 21° 12' E	679	132	61	9.5	1.5	0.655	0.590	0.091
	Galggojavri	69° 07' N, 20° 46' E	501	348	46	3.2	0.2	0.453	0.455	-0.004
	Gallajavri	69° 05' N, 20° 54' E	596	167	48	6.7	0.6	0.674	0.657	0.024
	Kilpisjärvi	69° 01' N, 20° 50' E	473	3733	48	7.4	1.9	0.653	0.588	0.152
Pond										
	Leenanlampi	69° 05' N, 20° 52' E	650	0.14		75	2.4	0.1	0.359	0.338	0.056

*N*, number of samples; *Ar*, allelic richness; *H*_E_, expected heterozygosity; *H*_O_, observed heterozygosity.

To estimate adult population size in Leenanlampi, a mark-recapture study was performed using two 40 m long and 5 m high gill nets (mesh size, 10–20 mm) in 2010 (two days in August). Adult population size was estimated according to Chapman's low-bias modification of the Petersen’s estimator [31]. In short, the total number of adult charr (*N*) was estimated as follows: *N* = [(*n*_1_ + 1)(*n*_2_ + 1) / (*m* + 1)]– 1, where *n*_1_ and *n*_2_ are the numbers of charr in catches of day 1 and 2 respectively, and *m* is the number of charr recaptured. In addition, immigration and emigration between Galggojavri and Leenanlampi were investigated in the 4.3 km long creek connecting them. The creek is about 3 m wide and 20 cm deep when it drains from Leenanlampi, but it becomes narrower (20–100 cm) and shallower (2–10 cm) soon after. The average channel slope is 3.5 cm/m (150 m of altitudinal difference). The creek was completely blocked with a net fence (mesh size, 1.5 mm) near the pond entrance (about 400 m from the pond) for the whole summers in 2011–2013. The width of the creek at the gate site was about 1 m. Fish traps (mesh size, 6–10 mm) were located on both sides of the mesh gate, and nets (mesh size, 10 mm) were set to guide fish into the traps. The fence and traps were checked at least three times a week, and the number of Arctic charr on either side of the mesh gate was counted. Immigrants and emigrants were evaluated based on the fish found at the Galggojavri and Leenanlampi sides, respectively. The fish collected at the Galggojavri side (i.e. immigrants) were photographed and released to the other side to see if they reach to Leenanlampi. They were identified based on size and shape and were distinguishable from emigrants from Leenanlampi as their body lengths were much bigger than those of Leenanlampi fish. We were particularly interested in examining if they can pass a stony stretch near Leenanlampi where they need to climb 1.3 m against a water flow of 1–2 m/s. The fish caught from the Leenanlampi side were released on the Galggojavri side. Since their adipose fins were clipped earlier in the sampling and mark-recapture study, they were distinguished from “true immigrants” from Galggojavri based on adipose fins.

### Microsatellite genotyping

DNA was extracted from fin clips or muscle tissue with silica-based methods [32,33] or a Chelex-based protocol [34]. Microsatellite analyses were performed using 15 loci: Str73 [35], Sfo-8, Sfo-23 [36], Str85INRA [37], Ssa-85 [38], One11ASC [39], Sco19SFU [40], Smm-17, Smm-24 [41], Sco200, Sco202, Sco204, Sco205, Sco213 and Sco218 [42]. The 5′-end of each reverse primer was modified with a GTTT-tail [43]. The 15 loci were arranged in multiplex PCR panels with non-overlapping size ranges in each dye. PCR was conducted in a 10 μl volume containing 5 μl of 2× Phusion Flash Master Mix (Finnzymes), 2 ρmol of each primer and approximately 10 ng of DNA. The reactions were performed with the following cycle profile: 98°C for 1 min, 34 cycles of 98°C for 1 s, 58°C for 15 s and 72°C for 20 s, and 72°C for 1 min. The PCR amplicons were analyzed using an ABI 3730 sequencer (Applied Biosystems) with the GeneScan 500 ROX size standard (Applied Biosystems). Alleles were scored using GeneMapper v.4.1 (Applied Biosystems).

### Data analyses

Expected heterozygosity, *F*_IS_ and *F*_ST_ [44] were calculated using FSTAT 2.9.3.2 [45]. The standard error of *F*_ST_ was obtained by jackknifing over loci. Allelic richness and private allelic richness were estimated using HP-RARE 1.1 [46] with a rarefaction sample size of 26 individuals. Deviations from Hardy-Weinberg equilibrium were assessed using exact tests (10,000 dememorization steps, 20 batches, 5000 iterations per batch) with GENEPOP 4.0.7 [47]. Bonferroni corrections were applied for all multiple comparisons. Comparisons of genetic diversity measures (i.e. heterozygosity and allelic richness) were conducted using one-way ANOVAs using population as a factor, followed by post-hoc tests (Fisher’s PLSD) for pairwise population comparisons. Pearson product moment correlations were used to test for associations between genetic variability measures and environmental variables. All statistical analyses were conducted using SPSS 13.0 (SPSS Inc.).

Recent population bottlenecks were inferred using BOTTLENECK 1.2 [48], assuming a two-phase mutation model (90% stepwise and 10% infinite models). This analysis tests for a relative heterozygote excess that is apparent for a few generations after a population bottleneck. Statistical significance was assessed by the one-tailed Wilcoxon signed-rank test with 10,000 iterations. Since One11ASC and Sco200 loci did not fit a step-wise mutation model in Galggojavri, these loci were excluded from the analyses of this population. In addition, population bottlenecks for longer periods of time (>100 generations) were investigated using Garza and Williamson’s *M* statistic, which is the mean ratio between the number of alleles and the allelic range [49]. The analysis was performed using M_P_VAL (https://swfsc.noaa.gov/textblock.aspx?Division=FED&id=3298). The mean number of non-stepwise mutations was set as 0.10 and the mean size of larger mutation as 3.5. Theta was set to the value estimated by MIGRATE for each population. The critical value for *M* (*M*_c_) for each population was calculated using CRITICAL_M (https://swfsc.noaa.gov/textblock.aspx?Division=FED&id=3298). For the estimation of *M*, monomorphic loci, as well as the loci that did not follow step-wise mutation model, were removed from the analysis of each population. Contemporary effective population size was estimated using the linkage disequilibrium method [50] implemented in LDNE [51].

Isolation by distance was analyzed by correlating the pairwise genetic differentiation measured by *F*_ST_/(1 –*F*_ST_) with the logarithm of the geographical distance between the populations using the Mantel’s test (1000 permutations) as implemented in GENEPOP. The possible impact of altitudinal differences on genetic differentiation among the populations was assessed using the Mantel’s test. The significance of genetic differentiation between the populations of different drainages (i.e. Atlantic Ocean and Baltic Sea) was examined using the Mantel’s test and analysis of molecular variance (AMOVA) [52]. The AMOVA (1000 permutations) was performed by grouping the populations into the different drainages with ARLEQUIN 3.5 [53]. Genetic relationships among populations were assessed using *D*_A_ distances [54], which provide better accuracy of tree topology than other distance measures irrespective of the presence or absence of population bottleneck effects [55]. A neighbor-joining (NJ) tree was constructed by bootstrapping (1000 replicates) across loci using POPULATIONS 1.2 [56]. Genetic population structure was also investigated using a Bayesian approach implemented in STRUCTURE 2.2 [57]. The analysis was performed using an admixture model of correlated allele frequencies with 50,000 burn-in length periods and 100,000 MCMC repetitions. Ten parallel chains were run for each of *K* = 1–9. The number of clusters (*K*) was determined based on the log likelihood and Δ*K* [58]. We also conducted a Bayesian admixture analysis implemented in BAPS 6.0 [59] in order to infer individuals of mixed ancestry. The admixture coefficient was estimated for the partitions inferred from the STRUCTURE analysis with recommended settings (100 iterations per individual, 200 reference individuals and 20 iterations per reference individual). The significance of admixture was determined based on a *P* value in each individual.

Historical (inbreeding) effective population size and migration rate were analyzed simultaneously using the maximum likelihood coalescent approach implemented in MIGRATE 3.6 [60]. Theta (θ = 4*N*_*e*_μ, where *N*_*e*_ is effective population size and μ mutation rate) and the migration parameter *M* (*m*/μ, where *m* is migration rate) were estimated under a stepwise mutation model with a Markov chain Monte Carlo (MCMC) repetition of 20 short chains of 20,000 steps and three long chains of 200,000 steps. *F*_ST_-based estimates were used as the starting parameters, and the burn-in was set to 10,000. The Gelman’s convergence criterion was applied to extend the long chains until the criterion was satisfied. The parameter estimates were obtained by combining five independent runs.

## Results

### Genetic variation

In total, 246 alleles were detected in six populations across 15 loci, with an average of 16.4 alleles per locus (S1 Table). Among the 15 loci, deviations from Hardy-Weinberg equilibrium were indicated for six loci in at least one population (S1 Table). Since two loci (Sfo-23 and Sco205) exhibited significantly positive *F*_IS_ values in two populations possibly due to the presence of null alleles, these loci were excluded from further analyses.

Among the six populations, average allelic richness and expected heterozygosity varied from 2.4 to 9.7 (ANOVA, *F*_5,72_ = 9.45, *P* < 0.001) and from 0.359 to 0.674 (ANOVA, *F*_5,72_ = 3.06, *P* = 0.015), respectively (Table 1). Leenanlampi showed the lowest values in both parameters (Fisher’s PLSD, *P* < 0.05 with all populations except for Galggojavri). Similarly, relatively low estimates were observed in Galggojavri. No significant correlation was observed between lake size and allelic richness (*r* = 0.257, *N* = 6, *P* = 0.623) or expected heterozygosity (*r* = 0.309, *N* = 6, *P* = 0.551). Likewise, there was no significant correlation neither between altitude and allelic richness (*r* = 0.209, *N* = 6, *P* = 0.691) nor between altitude and expected heterozygosity (*r* = 0.088, *N* = 6, *P* = 0.868). Private allelic richness ranged from 0.1 to 1.9 among the populations (Table 1). In the BOTTLENECK analysis, evidence for recent population bottlenecks was not detected in any populations (Table 2), although the probability value was close to significance in Leenanlampi (*P* = 0.065). In contrast, in the analysis of Garza and Williamson’s statistic, *M* values were lower than the critical values of *M* (*M*_c_) in all populations except Urtas-Riimmajärvi (Table 2), indicating genetic bottlenecks in the more remote past.

**Table 2:** Estimates of population bottleneck and effective population size (*N*_e_) in the six Arctic charr populations.

Population	BOTTLENECK	*M* statistic	LDNE	MIGRATE	
		*M (M*_c_ *)*	*N*_e_ (95% C.I.)	θ (95% C.I.)	*N*_e_ (95% C.I.)
Somasjärvi	*P* = 0.575	0.687 (0.795)	88.3 (62.6–141.3)	0.93 (0.87–1.00)	466 (437–498)
Urtas-Riimmajärvi	*P* = 0.997	0.779 (0.765)	227.6 (138.5–574.6)	1.09 (1.02–1.16)	543 (510–579)
Galggojavri	*P* = 0.213	0.520 (0.800)	12.1 (8.3–17.3)	0.49 (0.46–0.52)	243 (230–258)
Gallajavri	*P* = 0.212	0.691 (0.788)	97.0 (63.3–187.3)	0.80 (0.76–0.86)	402 (378–428)
Kilpisjärvi	*P* = 0.998	0.627 (0.793)	71.6 (51.5–111.0)	0.69 (0.64–0.74)	344 (322–368)
Leenanlampi	*P* = 0.065	0.616 (0.833)	7.0 (3.7–10.8)	0.22 (0.21–0.23)	108 (103–114)

The contemporary effective population sizes estimated using LDNE ranged from 7.0 to 227.6 among the populations (Table 2). A significant correlation was found between the estimates of contemporary effective population size and allelic richness (*r* = 0.908, *N* = 6, *P* = 0.012), but not between the former and lake size (*r* = -0.087, *N* = 6, *P* = 0.874).

### Genetic differentiation

**Fig 2:**
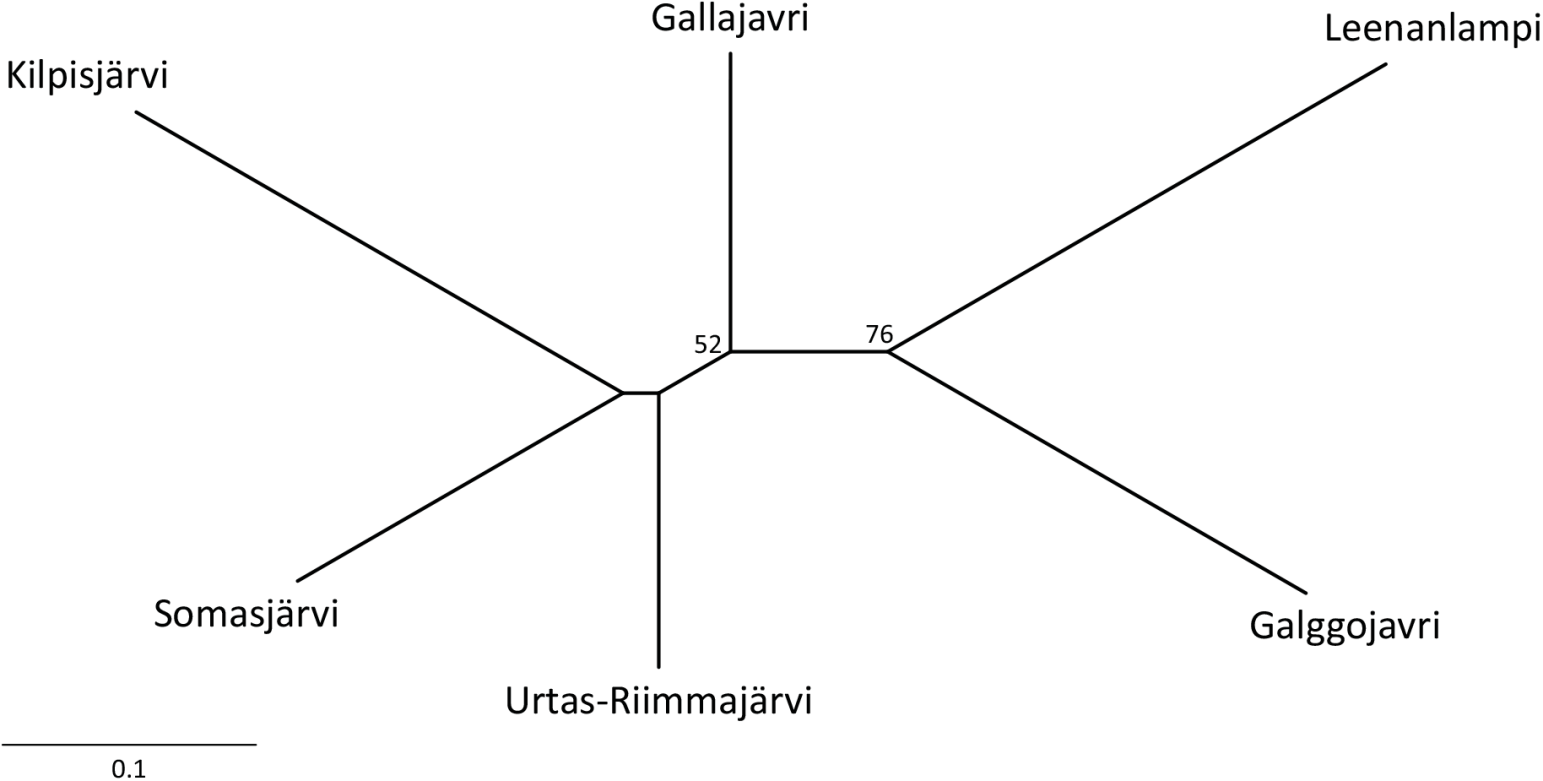
An unrooted neighbor-joining tree based on *D*_A_ distances among the six Arctic charr populations. Bootstrap support (>50%) is given at each node.

**Fig 3:**
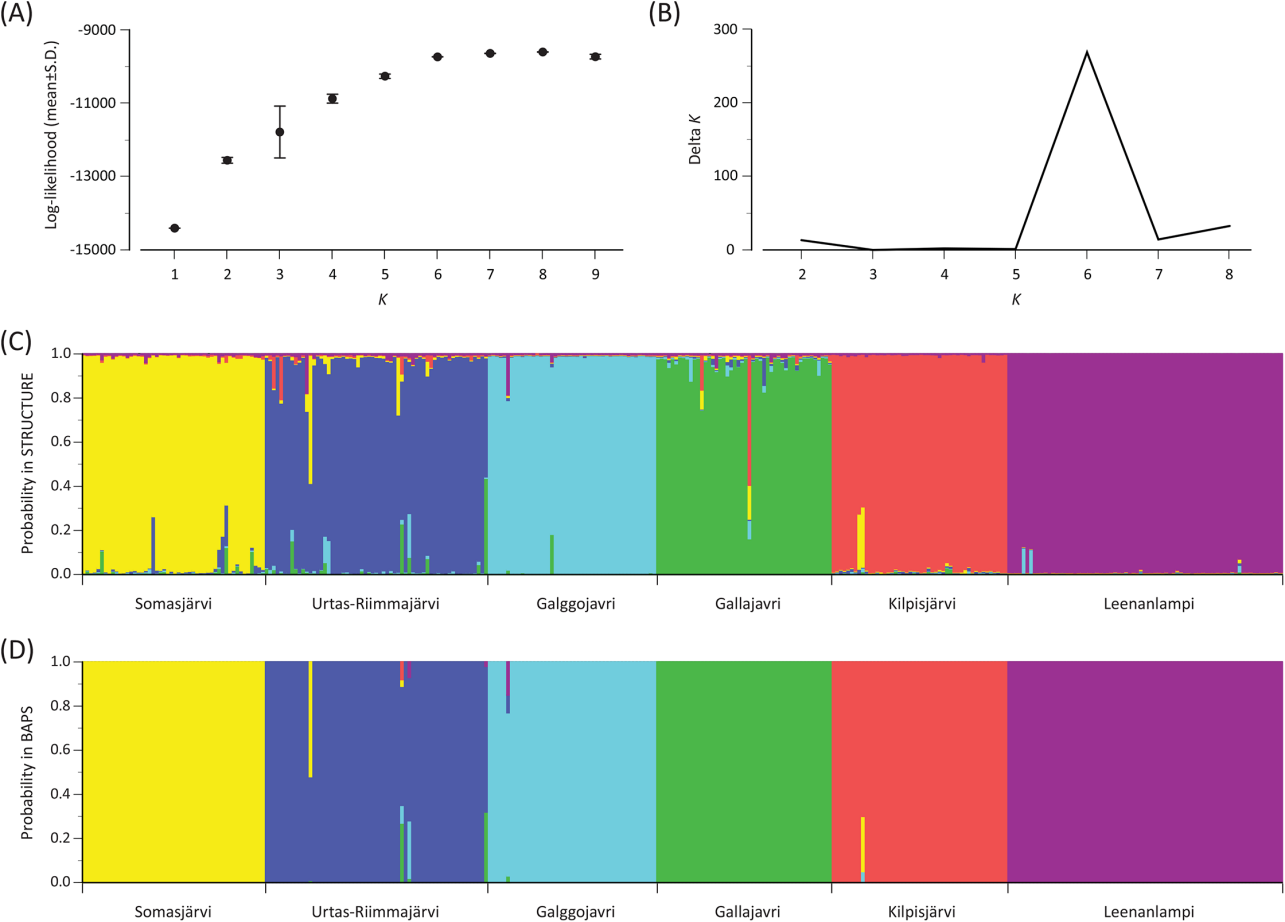
Bayesian clustering analyses for the six Arctic charr populations based on STRUCTURE and BAPS. (A) Likelihood estimates for *K* = 1–9 in STRUCTURE. (B) Estimated delta *K* for *K* = 2–8 in STRUCTURE. (C) Individual assignment at *K* = 6 in STRUCTURE. (D) Individual assignment at *K* = 6 in BAPS. Each individual is shown in a vertical bar in the same sequence (C and D).

The average *F*_ST_ for the six populations was 0.257 (S.E. = 0.033), with pairwise *F*_ST_ estimates ranging from 0.122 to 0.437 (Table 3). Neither a significant pattern of isolation by distance (*P* = 0.811) nor an association between pairwise *F*_ST_ and altitudinal differences (*P* = 0.668) was observed among the populations. Likewise, there was no correspondence between genetic differentiation and drainages (cf. Atlantic Ocean or Baltic Sea) of the populations as assessed by Mantel’s test (*P* = 0.510) or by between drainage component from AMOVA (*F*_CT_ = 2.73%, *P* = 0.109). The NJ tree constructed based on *D*_A_ distances (Table 3) showed a high degree of subdivision among the populations. A relatively high bootstrap support (76%) was obtained for the clustering of Galggojavri and Leenanlampi (Fig 2). In the STRUCTURE analysis, the log likelihood became saturated at *K* = 6 where a clear peak of Δ*K* was detected, implying the presence of six genetic clusters (Fig 3A and 3B). Each of the clusters consisted mostly of individuals from one population only, although the membership coefficient was less than 70% in seven out of the 328 individuals (Fig 3C; see also S2 Table). In the BAPS analysis, significant probabilities (*P* < 0.05) of admixture were found for six individuals, including four in Urtas-Riimmajärvi, one in Galggojavri and one in Kilpisjärvi (Fig 3D). The individuals of Galggojavri and Kilpisjärvi showed the highest proportions of admixture from Leenanlampi and Somasjärvi, respectively. In the individuals of Urtas-Riimmajärvi, the highest proportion of admixture derived from Somasjärvi, Galggojavri or Gallajavri.

**Table 3:** Pairwise *F*_ST_ (±S.E.) estimates (lower diagonal) and *D*_A_ distances (upper diagonal) among the six Arctic charr populations.

Population	Somasjärvi	Urtas-Riimmajärvi	Galggojavri	Gallajavri	Kilpisjärvi	Leenanlampi
Somasjärvi	-	0.287	0.508	0.339	0.400	0.520
Urtas-Riimmajärvi	0.142 ± 0.035	-	0.424	0.295	0.378	0.436
Galggojavri	0.293 ± 0.048	0.268 ± 0.042	-	0.368	0.574	0.451
Gallajavri	0.122 ± 0.031	0.122 ± 0.027	0.225 ± 0.039	-	0.401	0.472
Kilpisjärvi	0.149 ± 0.030	0.144 ± 0.028	0.313 ± 0.045	0.133 ± 0.028	-	0.602
Leenanlampi	0.347 ± 0.061	0.309 ± 0.067	0.437 ± 0.075	0.317 ± 0.057	0.363 ± 0.048	-

### Historical effective size and migration rate

The theta estimated by MIGRATE, which is an indicative of historical effective population size, ranged from 0.22 to 1.09 among the populations (Table 2). Relatively small values were observed in Leenanlampi (0.22) and Galggojavri (0.49). Assuming a microsatellite mutation rate of 5 × 10^−4^ [61,62], the historical effective population sizes were estimated to range from 108 to 543 depending on the population (Table 2). Historical and contemporary effective population size estimates obtained with MIGRATE and LDNE were strongly and positively correlated (*r* = 0.879, *N* = 6, *P* = 0.021).

The estimates of historical migration parameter (*M*) varied from 0.28 to 6.10 among the population pairs (Table 4). These values correspond to migration rates of 0.0001 to 0.0031 assuming a mutation rate of 5 × 10^−4^. The estimates of *M* from Somasjärvi to Urtas-Riimmajärvi and vice versa were relatively high (6.01 and 6.10, respectively). The *M* values estimated from these populations to the remaining four populations were smaller (0.55–3.96) than those obtained in the opposite directions (0.80–4.65) in some of the respective pairs (Table 4). The *M* values were relatively high from Gallajavri to Somasjärvi (4.65) and Urtas-Riimmajärvi (3.73). Relatively small *M* estimates were observed between Leenanlampi and Galggojavri (0.75 and 1.03) and between Leenanlampi and Kilpisjärvi (0.61 and 0.28).

**Table 4:** Estimates of historical migration rates (*M*; 95% C.I.) among the six Arctic charr populations.

	Population (to)					
Population (from)	Somasjärvi	Urtas-Riimmajärvi	Galggojavri	Gallajavri	Kilpisjärvi	Leenanlampi
Somasjärvi	-	6.01 (5.40–6.66)	0.74 (0.54–0.98)	2.34 (1.97–2.74)	1.42 (1.12–1.77)	0.72 (0.51–1.00)
Urtas-Riimmajärvi	6.10 (5.47–6.77)	-	0.55 (0.38–0.75)	3.96 (3.49–4.48)	1.87 (1.53–2.26)	1.43 (1.10–1.81)
Galggojavri	0.92 (0.69–1.20)	1.79 (1.47–2.15)	-	3.44 (3.00–3.93)	1.78 (1.45–2.16)	1.03 (0.76–1.36)
Gallajavri	4.65 (4.10–5.23)	3.73 (3.26–4.25)	2.64 (2.25–3.07)	-	2.36 (1.98–2.80)	2.21 (1.81–2.67)
Kilpisjärvi	1.04 (0.79–1.33)	2.19 (1.83–2.59)	1.47 (1.18–1.81)	2.01 (1.68–2.39)	-	0.28 (0.15–0.46)
Leenanlampi	0.80 (0.59–1.06)	3.36 (2.91–3.86)	0.75 (0.55–1.00)	1.48 (1.20–1.81)	0.61 (0.43–0.85)	-

### Field data

Based on mark-recapture data, the estimate of adult population size of Leenanlampi was 183 individuals (95% Poisson confidence interval, 157–212). While this estimate might be biased due to a low number (2) of recaptures, it corresponded to the total number (*N* = 184) of adult individuals caught in 2010–2012. Approximately 40 young-of-the-year individuals (about 5 cm in TL in early August) were observed in the pond, although the real number might be closer to 100. Therefore, including juveniles, the total late summer census population size was considered to be approximately 300 individuals.

The number of immigrants to Leenanlampi was 0, 1 and 1 individuals in 2011, 2012 and 2013, respectively. The corresponding figures for emigrants were 4, 3 and 10 individuals. The two immigrants were large (TL, 25 and 29 cm) and, therefore, probably effective migrants, whereas all emigrants were small (TL, 10–15 cm). However, both immigrants were unable to reach the pond itself, even though they tried to do so for several weeks, due to stony rapids and/or low water level in the last 400 m stretch connecting Leenanlampi to the creek.

## Discussion

The most salient findings of this study were the extremely small effective population size and the almost total lack of gene flow to and from the Leenanlampi pond population as inferred from molecular markers, which were concurrent with direct estimates of immigration and emigration rates and census population size obtained with ecological methods. We also showed that the contemporary genetic diversity of the northern Fennoscandian populations has been strongly impacted by historical demography rather than lake size. The high degree of genetic differentiation among all six study populations suggests that all of them are genetically and demographically effectively independent from each other. This aligns with the results of earlier population genetic studies of this species showing a high degree of genetic differentiation among local populations (e.g. [17,19,22]). In the following, we discuss each of these points in more in detail, as well as reflect upon the conservation implications of our findings.

### Genetic differentiation

There are numerous genetic studies of Arctic charr focused on both global [14,16] and more local scale diversity and divergence [19,20,22,63,64]. In line with the results of these earlier studies, we also detected a high degree of population differentiation, but little geographic structuring to this differentiation. Likewise, no isolation-by-distance was observed among the populations. The high level of divergence among local populations in our study is understandable in the light of two facts. First, the study populations are isolated, and thus there is little opportunity for gene flow among them. Although the localities within the watercourses are connected via pristine rivers, steep rapids and long distances likely prevent gene flow as the Arctic charr is not as efficient a swimmer as other salmonids [13]. Second, as indicated by our analyses of historical and contemporary effective population sizes, the effective population sizes are–and have been–fairly small even in the largest lake, subjecting populations to strong influence from genetic drift. The genetic divergence between the two geographically most closely situated study populations (Leenanlampi and Galggojavri), which are also connected by a small river, are a case in point. As indicated by both genetic and field data (see below), gene flow between these localities is very low, and the contemporary effective population size of the Leenanlampi population was estimated at *N*_e_ = 7. Hence, lack of gene flow together with small population sizes is likely to have caused the observed divergence among populations.

The lack or extremely low levels of gene flow between Leenanlampi and Galggojavri populations is supported by our field data. During three summers of exhaustive observations, little emigration and even less immigration to the Leenanlampi population was observed. The lack of immigration is understandable in light of the fact that only ≥ 25 cm long charr can pass rapids with 1 m/s water velocity [65]. In the creek connecting Leenanlampi to Galggojavri, there are many rapids with water flows 1–2 m/s even in midsummer. Furthermore, the upper reaches of the creek are shallow, in some places only a couple of centimeters deep, and large charr (a 25-cm long charr has a body depth of 5 cm) cannot efficiently pass them at all water levels. Thus, immigrants from Galggojavri are probably able to reach Leenanlampi only in exceptionally favorable conditions when there is enough water in the creek, but water flow is not too strong. It should be also noted that between late September and late May, the creek is completely frozen. Hence, all these considerations seem to support the genetic data which suggest a very low rate of gene flow to the Leenanlampi population. A rough calculation of effective number of immigrants per generation based on genetic data illustrates this. According to the equation *Nm* = [(1/*F*_ST_)– 1]/4 [66] with the *F*_ST_ value of 0.44, one arrives at an estimate of 0.3 individuals/generation. Based on the field data covering three years (ca. 0.6 charr generations), we observed zero individuals/generation. However, assuming that one charr generation equals five years, in light of the genetic data we would expect to observe one migrant only every 16.6 years.

### Genetic relationships and colonization history

Although the study populations reside at a watershed divide, three lakes currently discharging into the Baltic Sea used to discharge into the Atlantic Ocean [30]. Out of the three lakes, Somasjärvi and Urtas-Riimmajärvi were part of the same ice-dammed lake during the retreat of glacial ice [67]. Thus, the relatively high historical migration rates between these lakes could be due to their shared hydrogeographic history. In the study area, these lakes were free from ice first [67]. However, given the low historical migration rates from these populations to the others, it is unlikely that Arctic charr colonized this area via these lakes. Additionally, due to high altitude and difficult terrain, it is highly uncertain whether charr were able to ascend to Somasjärvi and Urtas-Riimmajärvi from the Atlantic Ocean. In contrast, the relatively high historical migration rates from Gallajavri to these lakes imply that colonization and gene flow might have occurred in this direction in the past. It is also possible that, when the present drainage system was formed, Arctic charr colonized this area via the current watercourse connection (ca. 9500 years BP). Postglacial colonization of the northernmost parts of Finland and Norway from an eastern refugium has been observed for European whitefish (*Coregonus lavaretus*) [68], grayling (*Thymallus thymallus*) [69], nine-spined stickleback (*Pungitius pungitius*) [70,71] and perch (*Perca fluviatilis*) [72]. However, we found no clear evidence regarding the colonization history of the study populations. The amount of genetic drift these populations have experienced is likely to have contributed to the lack of resolution in the phylogeographic analysis. Furthermore, the inability of fast evolving microsatellite loci to resolve phylogenetic relationships among populations that diverged from each other several thousands of years ago is a well-known problem [73,74]. Given the complex geological and hydrological history of this region [28,30,67,73,75–77], as well as genetic drift, it is difficult to draw firm conclusions about the phylogeographical relationships and colonization history based on our data. Nevertheless, it appears that the contemporary genetic structure has been largely affected by historical factors, as evidenced by the presence of historical (but not recent) population bottlenecks, as well as the strong correlation between the historical and contemporary effective population sizes (see also below).

The clustering analyses identified possible admixtures in a small proportion of individuals in some of the study localities, although none of them are known to be subject to stocking or farming activities. The possible introgressed individuals in Galggojavri and Kilpisjärvi were indicated to have been admixed with the ancestors of Leenanlampi and Somasjärvi, respectively. Since these admixtures were found within the same watercourses, migration might have taken place via the watercourses. Similarly, a possible admixture of Somasjärvi into Urtas-Riimmajärvi was observed within the same watercourse. However, other possible introgressed individuals in Urtas-Riimmajärvi were indicated to have admixed with the ancestors of Galggojavri and Gallajavri currently belonging to a different watercourse. Since it is unlikely that migration could take place from these lakes to Urtas-Riimmajärvi, the possible admixtures might result from artificial transfers by indigenous fishermen in the past, although no such records are available. It should be also noted that the accuracy of clustering and admixture analyses largely depends on the number of markers utilized [78,79]. Given that the number of markers investigated in our study is much smaller than the chromosome number (2n = 78) of this species [80], it cannot be ruled out that our genetic data might have insufficient power to identify genetic introgression accurately.

### Effective population size

Despite the difficulty of estimating effective population size (*N*_e_) with precision and without bias, it can provide a useful proxy for predicting population viability and fitness (e.g. [81,82]). In this study, we estimated both historical and contemporary effective sizes in six different Arctic charr populations, and found that the estimates were strongly positively correlated. It is noteworthy that this correlation has been rarely observed in empirical studies, suggesting that the influence of historical and contemporary effects on genetic diversity of populations covary rarely (e.g. [83–85]). While these two sets of estimates are not entirely independent as they were estimated from the same data, they may nevertheless suggest some degree of temporal consistency in genetically effective sizes of Arctic charr populations since their establishment after the last glaciations. Perhaps more interestingly, for all of the six populations, the upper confidence intervals for point estimates of contemporary *N*_e_ were below 600. In most cases the estimates suggested effective sizes around 100 or less (average = 84). While these values are very low, especially in the case of the Leenanlampi population with *N*_e_ = 7 (95% C.I. = 4–11), they are well within the range of previously reported values from a wide range of taxa [81]. Applying the conservative thresholds for critical population size [86,87], all but perhaps one of the six study populations appear to lack sufficient evolutionary potential for long-term evolution (i.e. *N*_e_ < 500). In addition, two of the populations with *N*_e_ < 50 may also suffer from adverse effects of inbreeding depression.

Leenanlampi population provides an interesting benchmarking case because of the availability of data on the census population size (*N*_C_). By conducting an exhaustive mark-recapture study, we estimated the size of the census population to be around 300 individuals. This translates to *N*_e_/*N*_C_-ratio of 0.02, which is much lower than the median value (0.15) of 66 studies reviewed in Palstra and Ruzzante [82]. If we use this ratio to estimate the census population sizes in the other lakes with *N*_e_ = 12–228, we obtain *N*_C_ = 519–9754 individuals. However, as discussed in Palstra and Ruzzante [82], it is unclear whether *N*_e_/*N*_C_-ratios can be considered temporally constant, and hence, caution should be exercised when estimating *N*_e_ from *N*_C_ (or vice versa).

Finally, it is worth noting that many of the population genetic parameters estimated in this study were rather reliable, including the estimates of contemporary effective population sizes which are notoriously difficult to estimate with any precision [82]. Although perhaps by no means high according to standards of many current genomic approaches, the relatively large number of loci (*N* = 13) and average number (55) of genotyped individuals per population utilized in this study certainly contributed to this. These figures are amongst the highest used in population genetic studies of Arctic charr: typically ≤10 microsatellite markers have been utilized. Nevertheless, an even larger panel of markers–as obtainable for instance by using high-throughput sequencing (e.g. [88])–and sampling of larger geographical area are required to resolve conclusively the phylogenetic relationships among the populations, and the colonization history.

## Conclusions

Our study uncovered a high degree of genetic differentiation among the northern Fennoscandian Arctic charr populations on a very small geographical scale. This differentiation is understandable in light of very limited gene flow and strong genetic drift. Although some of the study populations are connected by pristine rivers and brooks, there is little or no evidence for ongoing gene flow between them neither from genetic or field data. In addition, our results suggest a strong impact of historical factors on the contemporary genetic diversity and effective population size. Given the low genetic variability and small effective size of the Leenanlampi population, it appears to be vulnerable to further loss of genetic variability and may have limited capacity to adapt on changing selection pressures.

## Supporting Information

S1 Table Genetic variation at 15 microsatellite loci in the six Arctic charr populations. (XLSX)

S2 Table Mean (±95% C.I.) of membership coefficient in STRUCTURE analysis in the six Arctic charr populations. (XLSX)

## References

[1] DeWoody JA, Avise JC (2000) Microsatellite variation in marine, freshwater and anadromous fishes compared with other animals. J Fish Biol 56: 461–473.

[2] Merilä J (2014) Lakes and ponds as model systems to study convergent and parallel evolution. J. Limnol 73: 33–45.

[3] Frankham R, Ballou JD, Briscoe DA (2002) Introduction to Conservation Genetics. Cambridge: Cambridge University Press.

[4] Hewitt G (2000) The genetic legacy of the Quaternary ice ages. Nature 405: 907–913. 10879524 10.1038/35016000

[5] Hewitt GM (2004) The structure of biodiversity–insights from molecular phylogeography. Front Zool 1: 1–16. 15679920 10.1186/1742-9994-1-4 PMC544936

[6] Reed DH, Frankham R (2001) How closely correlated are molecular and quantitative measures of genetic variation? A meta‐analysis. Evolution 55: 1095–1103. 11475045 10.1111/j.0014-3820.2001.tb00629.x

[7] Caballero A, García-Dorado A (2013) Allelic diversity and its implications for the rate of adaptation. Genetics 195: 1373–1384. 10.1534/genetics.113.158410 24121776 PMC3832279

[8] Reed DH, Frankham R (2003) Correlation between fitness and genetic diversity. Conserv Biol 17: 230–237.

[9] Willi Y, Van Buskirk J, Hoffmann AA (2006) Limits to the adaptive potential of small populations. Ann Rev Ecol Evol Syst 37: 433–458.

[10] Kopp M, Matuszewski S (2014) Rapid evolution of quantitative traits: theoretical perspectives. Evol Appl 7: 169–191. 10.1111/eva.12127 24454555 PMC3894905

[11] Jonsson B, Jonsson N (2001) Polymorphism and speciation in Arctic charr. J Fish Biol 58: 605–638.

[12] Klemetsen A (2013) The most variable vertebrate on earth. J Icthyol 53: 781–791.

[13] Klemetsen A, Amundsen PA, Dempson JB, Jonsson B, Jonsson N, O'Connell MF, et al (2003) Atlantic salmon *Salmo salar* L., brown trout *Salmo trutta* L. and Arctic charr *Salvelinus alpinus* (L.): a review of aspects of their life histories. Ecol Freshw Fish 12: 1–59.

[14] Brunner PC, Douglas MR, Osinov A, Wilson CC, Bernatchez L (2001) Holarctic phylogeography of Arctic charr (*Salvelinus alpinus* L.) inferred from mitochondrial DNA sequences. Evolution 55: 573–586. 11327164 10.1554/0014-3820(2001)055[0573:hpoacs]2.0.co;2

[15] Hindar K, Ryman N, Ståhl G (1986) Genetic differentiation among local populations and morphotypes of Arctic charr, *Salvelinus alpinus* . Biol J Linn Soc 27: 269–285.

[16] Wilson CC, Hebert PDN, Reist JD, Dempson JB (1996) Phylogeography and postglacial dispersal of arctic charr *Salvelinus alpinus* in North America. Mol Ecol 5: 187–197.

[17] Brunner PC, Douglas MR, Bernatchez L (1998) Microsatellite and mitochondrial DNA assessment of population structure and stocking effects in Arctic charr *Salvelinus alpinus* (Teleostei: Salmonidae) from central Alpine lakes. Mol Ecol 7: 209–223.

[18] Primmer CR, Aho T, Piironen J, Estoup A, Cornuet JM, Ranta E (1999) Microsatellite analysis of hatchery stocks and natural populations of Arctic charr, *Salvelinus alpinus*, from the Nordic region: implications for conservation. Hereditas 130: 277–289.

[19] Wilson AJ, Gislason D, Skulason S, Snorrason SS, Adams CE, Alexander G, et al (2004) Population genetic structure of Arctic charr, *Salvelinus alpinus* from northwest Europe on large and small spatial scales. Mol Ecol 13: 1129–1142. 15078451 10.1111/j.1365-294X.2004.02149.x

[20] Alekseyev SS, Bajno R, Gordeeva NV, Reist JD, Power M, Kirillov AF, et al (2009) Phylogeography and sympatric differentiation of the arctic charr *Salvelinus alpinus* (L.) complex in Siberia as revealed by mtDNA sequence analysis. J Fish Biol 75: 368–392. 10.1111/j.1095-8649.2009.02331.x 20738544

[21] Gordeeva NV, Osinov AG, Alekseyev SS, Matveev AN, Samusenok VP (2010) Genetic differentiation of Arctic charr *Salvelinus alpinus* complex from Transbaikalia revealed by microsatellite markers. J Ichthyol 50: 351–361.

[22] Kapralova KH, Morrissey MB, Kristjánsson BK, Ólafsdóttir GÁ, Snorrason SS, Ferguson MM (2011) Evolution of adaptive diversity and genetic connectivity in Arctic charr (*Salvelinus alpinus*) in Iceland. Heredity 106: 472–487. 10.1038/hdy.2010.161 21224880 PMC3131972

[23] Bernatchez L, Dempson JB, Martin S (1998) Microsatellite gene diversity analysis in anadromous arctic char, *Salvelinus alpinus*, from Labrador, Canada. Can J Fish Aquat Sci 55: 1264–1272.

[24] Moore JS, Harris LN, Tallman RF, Taylor EB (2013) The interplay between dispersal and gene flow in anadromous Arctic char (*Salvelinus alpinus*): implications for potential for local adaptation. Can J Fish Aquat Sci 70: 1327–1338.

[25] Maitland PS (1995) World status and conservation of the Arctic Charr *Salvelinus alpinus* L. Nord J Freshw Res 71: 113–127.

[26] Urho L, Pennanen JT, Koljonen ML (2010) Fish In: Rassi P, Hyvärinen E, Juslén A, Mannerkoski I, editors. The 2010 Red List of Finnish Species. Helsinki: Ympäristöministeriö & Suomen Ympäristökeskus pp. 336–343.

[27] Sollid JL, Andersen S, Hamre N, Kjeldsen O, Salvigsen O, Sturød S (1973) Deglaciation of Finnmark, North Norway. Nor Geogr Tidsskr 27: 233–325.

[28] Mangerud J, Jakobsson M, Alexanderson H, Astakhov V, Clarke GKC, Henriksen M, et al (2004) Ice-dammed lakes and rerouting of the drainage of northern Eurasia during the last glaciation. Quat Sci Rev 23: 1313–1332.

[29] Svendsen JI, Alexanderson H, Astakhov VI, Demidov I, Dowdeswell JA, Funder S, et al (2004) Late quaternary ice sheet history of northern Eurasia. Quat Sci Rev 23: 1229–1271.

[30] Johansson P, Kujansuu R (2005) Pohjois-Suomen Maaperä. Espoo: Geological Survey of Finland p. 236.

[31] Chapman DG (1948) A mathematical study of confidence limits of salmon populations calculated from sample tag ratios. Int Pac Salmon Fish Comm Bull 2: 67–85.

[32] Elphinstone MS, Hinten GN, Anderson MJ, Nock CJ (2003) An inexpensive and high-throughput procedure to extract and purify total genomic DNA for population studies. Mol Ecol Notes 3: 317–320.

[33] Ivanova NV, deWaard JR, Hebert PDN (2006) An inexpensive, automation-friendly protocol for recovering high-quality DNA. Mol Ecol Notes 6: 998–1002.

[34] Walsh PS, Metzger DA, Higuchi R (1991) Chelex 100 as a medium for simple extraction of DNA for PCR-based typing from forensic material. BioTechniques 10: 506–513. 1867860

[35] Estoup A, Presa P, Krieg F, Vaiman D, Guyomard R (1993) (CT)_n_ and (GT)_n_ microsatellites: a new class of genetic markers for *Salmo trutta* L. (brown trout). Heredity 71: 488–496. 8276633 10.1038/hdy.1993.167

[36] Angers B, Bernatchez L, Angers A, Desgroseillers L (1995) Specific microsatellite loci for brook charr reveal strong population subdivision on a microgeographic scale. J Fish Biol 47: 177–185.

[37] Presa P, Guyomard R (1996) Conservation of microsatellites in three species of salmonids. J Fish Biol 49: 1326–1329.

[38] O’Reilly PT, Hamilton LC, McConnell SK, Wright JM (1996) Rapid analysis of genetic variation in Atlantic salmon (*Salmo salar*) by PCR multiplexing of dinucleotide and tetranucleotide microsatellites. Can J Fish Aquat Sci 53: 2292–2298.

[39] Scribner KT, Gust JR, Fields RL (1996) Isolation and characterization of novel microsatellite loci: cross-species amplification and population genetic applications. Can J Fish Aquat Sci 53: 833–841.

[40] Taylor EB, Redenbach Z, Costello AB, Pollard SM, Pacas CJ (2001) Nested analysis of genetic diversity in northwestern North American char, Dolly Varden (*Salvelinus malma*) and bull trout (*Salvelinus confluentus*). Can J Fish Aquat Sci 58: 406–420.

[41] Crane PA, Lewis CJ, Kretschmer EJ, Miller SJ, Spearman WJ, DeCicco AL, et al (2004) Characterization and inheritance of seven microsatellite loci from Dolly Varden, *Salvelinus malma*, and cross-species amplification in Arctic char, *S alpinus* . Conserv Genet 5: 737–741.

[42] Dehaan PW, Ardren WR (2005) Characterization of 20 highly variable tetranucleotide microsatellite loci for bull trout (*Salvelinus confluentus*) and cross-amplification in other *Salvelinus* species. Mol Ecol Notes 5: 582–585.

[43] Brownstein MJ, Carpten JD, Smith JR (1996) Modulation of non-templated nucleotide addition by *Taq* DNA polymerase: primer modifications that facilitate genotyping. BioTechniques 20: 1004–1010. 8780871 10.2144/96206st01

[44] Weir BS, Cockerham CC (1984) Estimating *F*-statistics for the analysis of population structure. Evolution 38: 1358–1370. 28563791 10.1111/j.1558-5646.1984.tb05657.x

[45] Goudet J (1995) FSTAT (Version 1.2): a computer program to calculate F-statistics. J Hered 86: 485–486.

[46] Kalinowski ST (2005) HP-RARE 1.0: a computer program for performing rarefaction on measures of allelic richness. Mol Ecol Notes 5: 187–189.

[47] Rousset F (2008) Genepop'007: a complete reimplementation of the Genepop software for Windows and Linux. Mol Ecol Resour 8: 103–106. 10.1111/j.1471-8286.2007.01931.x 21585727

[48] Piry S, Luikart G, Cornuet JM (1999) BOTTLENECK: a computer program for detecting recent reductions in the effective population size using allele frequency data. J Hered 90: 502–503.

[49] Garza JC, Williamson EG (2001) Detection of reduction in population size using data from microsatellite loci. Mol Ecol 10: 305–318. 11298947 10.1046/j.1365-294x.2001.01190.x

[50] Waples RS (2006) A bias correction for estimates of effective population size based on linkage disequilibrium at unlinked gene loci. Conserv Genet 7: 167–184.

[51] Waples RS, Do C (2008) LDNE: A program for estimating effective population size from data on linkage disequilibrium. Mol Ecol Notes 8: 753–756. 10.1111/j.1755-0998.2007.02061.x 21585883

[52] Excoffier L, Smouse PE, Quattro JM (1992) Analysis of molecular variance inferred from metric distances among DNA haplotypes: application to human mitochondrial DNA restriction data. Genetics 131: 479–491. 1644282 10.1093/genetics/131.2.479 PMC1205020

[53] Excoffier L, Lischer HEL (2010) Arlequin suite ver 3.5: A new series of programs to perform population genetics analyses under Linux and Windows. Mol Ecol Resour 10: 564–567. 10.1111/j.1755-0998.2010.02847.x 21565059

[54] Nei M, Tajima F, Tateno Y (1983) Accuracy of estimated phylogenetic trees from molecular data. J Mol Evol 19: 153–170. 6571220 10.1007/BF02300753

[55] Takezaki N, Nei M (1996) Genetic distances and reconstruction of phylogenetic trees from microsatellite DNA. Genetics 144: 389–399. 8878702 10.1093/genetics/144.1.389 PMC1207511

[56] Langella O (2002) Populations 1.2.28. Logiciel de génétique des populations. Laboratoire Populations, Génétique et Evolution. Gif-sur-Yvette: CNRS UPR9034.

[57] Pritchard JK, Stephens M, Donnelly P (2000) Inference of population structure using multilocus genotype data. Genetics 155: 945–959. 10835412 10.1093/genetics/155.2.945 PMC1461096

[58] Evanno G, Regnaut S, Goudet J (2005) Detecting the number of clusters of individuals using the software structure: a simulation study. Mol Ecol 14: 2611–2620. 15969739 10.1111/j.1365-294X.2005.02553.x

[59] Corander J, Marttinen P, Sirén J, Tang J (2008) Enhanced Bayesian modelling in BAPS software for learning genetic structures of populations. BMC Bioinformatics 9: 539 10.1186/1471-2105-9-539 19087322 PMC2629778

[60] Beerli P, Felsenstein J (2001) Maximum likelihood estimation of a migration matrix and effective population sizes in n subpopulations by using a coalescent approach. Proc Natl Acad Sci USA 98: 4563–4568. 11287657 10.1073/pnas.081068098 PMC31874

[61] Estoup A, Angers B (1998) Microsatellites and minisatellites for molecular ecology: theoretical and empirical considerations In: Carvlho GR, editor. Advances in Molecular Ecology, NATO Science Series Amsterdam: IOS Press pp. 55–86.

[62] Lippe C, Dumont P, Bernatchez L (2006) High genetic diversity and no inbreeding in the endangered copper redhorse, *Moxostoma hubbsi* (Catostomidae, Pisces): the positive sides of a long generation time. Mol Ecol 15: 1769–1780. 16689897 10.1111/j.1365-294X.2006.02902.x

[63] Gíslason D, Ferguson MM, Skúlason S, Snorrason SS (1999) Rapid and coupled phenotypic and genetic divergence in Icelandic Arctic char (*Salvelinus alpinus*). Can J Fish Aquat Sci 56: 2229–2234.

[64] Bernatchez L, Rhydderch JG, Kircheis FW (2002) Microsatellite gene diversity analysis in landlocked Arctic char from Maine. Trans Am Fish Soc 131: 1106–1118.

[65] Sinnatamby RN, Babaluk JA, Power G, Reist JD, Power M (2012) Summer habitat use and feeding of juvenile Arctic charr, *Salvelinus alpinus*, in the Canadian High Arctic. Ecol Freshw Fish 21: 309–322.

[66] Wright S (1951) The genetic structure of populations. Ann Eugen 15: 313–354. 10.1111/j.1469-1809.1949.tb02451.x 24540312

[67] Kujansuu R (1967) On the deglaciation of western Finnish Lapland. Bull Comm Géol Finl 232: 1–98.

[68] Østbye K, Bernatchez L, Næsje TF, Himberg KJM, Hindar K (2005) Evolutionary history of the European whitefish *Coregonus lavaretus* (L.) species complex as inferred from mtDNA phylogeography and gill-raker numbers. Mol Ecol 14: 4371–4387. 16313599 10.1111/j.1365-294X.2005.02737.x

[69] Koskinen MT, Ranta E, Piironen J, Veselov A, Titov S, Haugen TO, et al (2000) Genetic lineages and postglacial colonization of grayling (*Thymallus thymallus*, Salmonidae) in Europe, as revealed by mitochondrial DNA analyses. Mol Ecol 9: 1609–1624. 11050556 10.1046/j.1365-294x.2000.01065.x

[70] Shikano T, Shimada Y, Herczeg G, Merilä J (2010) History vs. habitat type: explaining the genetic structure of European nine-spined stickleback (*Pungitius pungitius*) populations. Mol Ecol 19: 1147–1161. 10.1111/j.1365-294X.2010.04553.x 20163545

[71] Teacher AGF, Shikano T, Karjalainen ME, Merilä J (2011) Phylogeography and genetic structuring of European nine-spined sticklebacks (*Pungitius pungitius*)–mitochondrial DNA evidence. PLoS ONE 6: e19476 10.1371/journal.pone.0019476 21589917 PMC3092751

[72] Nesbø CL, Fossheim T, Vøllestad LA, Jakobsen KS (1999) Genetic divergence and phylogeographic relationships among European perch (*Perca fluviatilis*) populations reflect glacial refugia and postglacial colonization. Mol Ecol 8: 1387–1404. 10564445 10.1046/j.1365-294x.1999.00699.x

[73] Estoup A, Jarne P, Cornuet JM (2002) Homoplasy and mutation model at microsatellite loci and their consequences for population genetics analysis. Mol Ecol 11: 1591–1604. 12207711 10.1046/j.1365-294x.2002.01576.x

[74] Zhang DX, Hewitt GM (2003) Nuclear DNA analyses in genetic studies of populations: practice, problems and prospects. Mol Ecol 12: 563–584. 12675814 10.1046/j.1365-294x.2003.01773.x

[75] Tanner V (1907) Zur geologischen Geschichte des Kilpisjärvi-Sees in Lappland. Bull Comm Géol Finl 20: 1–23.

[76] Lundqvist J (1965) The quaternary of Sweden In: Rankama K, editor. The Geologic Systems: The Quaternary I. New York: Wiley pp. 139–198.

[77] Lundqvist J (1972) Ice-lake types and deglaciation pattern along the Scandinavian mountain range. Boreas 1: 27–54.

[78] Waples RS, Gaggiotti O (2006) What is a population? An empirical evaluation of some genetic methods for identifying the number of gene pools and their degree of connectivity. Mol Ecol 15: 1419–1439. 16629801 10.1111/j.1365-294X.2006.02890.x

[79] Porras-Hurtado L, Ruiz Y, Santos C, Phillips C, Carracedo A, Lareu MV (2013) An overview of STRUCTURE: applications, parameter settings, and supporting software. Front Genet 4: 98 10.3389/fgene.2013.00098 23755071 PMC3665925

[80] Phillips RB, Ráb P (2001) Chromosome evolution in the Salmonidae (Pisces): an update. Biol Rev 76: 1–25. 11325050 10.1017/s1464793100005613

[81] Reed DH (2005) Relationship between population size and fitness. Conserv Biol 19: 563–568.

[82] Palstra FP, Ruzzante DE (2008) Genetic estimates of contemporary effective population size: what can they tell us about the importance of genetic stochasticity for wild population persistence? Mol Ecol 17: 3428–3447. 19160474 10.1111/j.1365-294x.2008.03842.x

[83] Hansen MM, Skaala Ø, Jensen LF, Bekkevold D, Mensberg KLD (2007) Gene flow, effective population size and selection at major histocompatibility complex genes: brown trout in the Hardanger Fjord, Norway. Mol Ecol 16: 1413–1425. 17391266 10.1111/j.1365-294X.2007.03255.x

[84] Whiteley AR, Hastings K, Wenburg JK, Frissell CA, Martin JC, Allendorf FW (2010) Genetic variation and effective population size in isolated populations of coastal cutthroat trout. Conserv Genet 11: 1929–1943.

[85] Lotterhos KE, Dick SJ, Haggarty DR (2014) Evaluation of rockfish conservation area networks in the United States and Canada relative to the dispersal distance for black rockfish (*Sebastes melanops*). Evol Appl 7: 238–259. 10.1111/eva.12115 24567745 PMC3927886

[86] Franklin IR (1980) Evolutionary change in small populations In: Soule ME, Wilcox BA, editors. Conservation Biology: An Evolutionary-Ecological Perspective. Sunderland: Sinauer pp. 135–150.

[87] Franklin IR, Frankham R (1998) How large must populations be to retain evolutionary potential? Anim Conserv 1: 69–73.

[88] Emerson KJ, Merz CR, Catchen JM, Hohenlohe PA, Cresko WA, Bradshaw WE, et al (2010) Resolving postglacial phylogeography using high-throughput sequencing. Proc Natl Acad Sci USA 107: 16196–16200. 10.1073/pnas.1006538107 20798348 PMC2941283

